# A Low-Band Multi-Gain LNA Design for Diversity Receive Module with 1.2 dB NF

**DOI:** 10.3390/s21248340

**Published:** 2021-12-14

**Authors:** Behnam S. Rikan, David Kim, Kyung-Duk Choi, Seyed Ali H. Asl, Joon-Mo Yoo, YoungGun Pu, Seokkee Kim, Hyungki Huh, Yeonjae Jung, Kang-Yoon Lee

**Affiliations:** 1 Department of Electrical and Computer Engineering, Sungkyunkwan University, Suwon 16419, Korea; behnam@skku.edu (B.S.R.); dkim9402@skku.edu (D.K.); glyiiop@skku.edu (K.-D.C.); saha@skku.edu (S.A.H.A.); fiance2@g.skku.edu (J.-M.Y.); hara1015@skku.edu (Y.P.); seokkeekim@skku.edu (S.K.); gray.huh@skku.edu (H.H.); yj.jung@skku.edu (Y.J.); 2 SKAIChips Co., Ltd., Suwon 16419, Korea

**Keywords:** cascade with L degeneration, low-band LNA, multi-gain mode, phase discontinuity

## Abstract

This paper presents and discusses a Low-Band (LB) Low Noise Amplifier (LNA) design for a diversity receive module where the application is for multi-mode cellular handsets. The LB LNA covers the frequency range between 617 MHz to 960 MHz in 5 different frequency bands and a 5 Pole Single Throw (5PST) switch selects the different frequency bands where two of them are for the main and three for the auxiliary bands. The presented structure covers the gain modes from −12 to 18 dB with 6 dB gain steps where each gain mode has a different current consumption. In order to achieve the Noise Figure (NF) specifications in high gain modes, we have adopted a cascode Common-Source (CS) with inductive source degeneration structure for this design. To achieve the S_11_ parameters and current consumption specifications, the core and cascode transistors for high gain modes (18 dB, 12 dB, and 6 dB) and low gain modes (0 dB, −6 dB, and −12 dB) have been separated. Nevertheless, to keep the area low and keep the phase discontinuity within ±10${}^{\circ }$, we have shared the degeneration and load inductors between two cores. To compensate the performance for Process, Voltage, and Temperature (PVT) variations, the structure applies a Low Drop-Out (LDO) regulator and a corner case voltage compensator. The design has been proceeded in a 65-nm RSB process design kit and the supply voltage is 1 V. For 18 dB and −12 dB gain modes as two examples, the NF, current consumption, and Input Third Order Intercept Point (IIP3) values are 1.2 dB and 16 dB, 10.8 mA and 1.2 mA, and −6 dBm and 8 dBm, respectively.

## 1. Introduction

The recent movement toward higher-order Multi-Input Multi-Output (MIMO) Radio Frequency (RF) structures is compounding additional RF receive path support and cost-effective solutions for optimum performance trade-offs, where the diversity RF front-end structures have been proposed. Diversity RF front-end receiver module configuration includes a first antenna-side multi-throw switch, a second antenna-side multi-throw switch, a first RF front-end followed by a multi-throw switch, a second RF front-end followed by a multi-throw switch. A Low-Band (LB) signal path exists between the first antenna-side multi-throw switch and the first RF front-end. There are also Mid-High Band (MHB) signal paths between the second antenna-side multi-throw switch and the second RF front-end. These LB and MHB paths can operate concurrently [1,2].

**Figure 1:**
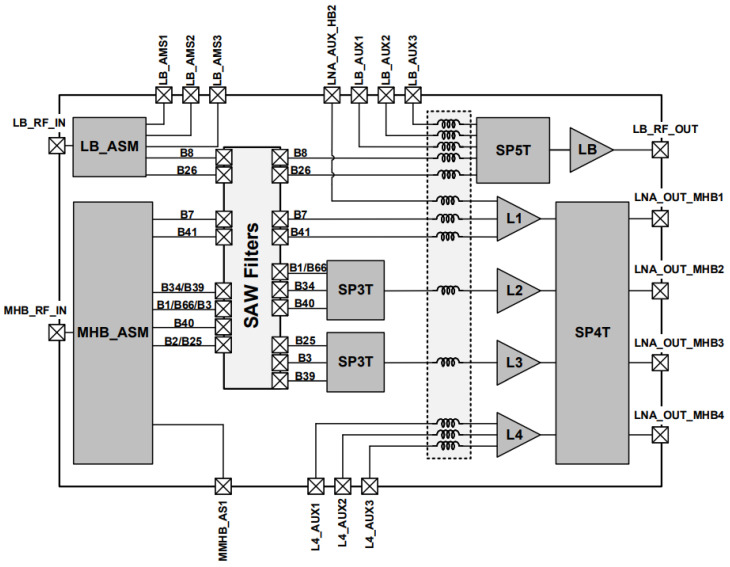
Block diagram of the diversity RF front-end for 2G/3G/4G/5G multi-mode cellular handsets.

Figure 1 presents the block diagram of the diversity RF front-end that can be applied in 2G/3G/4G/5G multi-mode cellular handsets (5G NR, LTE, UMTS, CDMA2000, EDGE, and GSM). This module that includes Surface Acoustic Wave (SAW) filters, support the following frequency bands: B8, B26, B3, B39, B25, B34, B66, B40, B41, B7, etc., and some auxiliary bands [3].

The focus of this paper is to implement the LB Low-Noise Amplifier (LNA) part where the frequency range is 617–960 MHz. This frequency range has been covered in five bands where B8 (925–960 MHz) and B26 (859–894 MHz) are the main bands of this design and are selected with two of the switches in 5 Pole Single Throw (5PST). Three auxiliary bands that are selected with the remaining three switches of the 5PST, cover the whole frequency range again including B8, B26 in one band, B71 & B29 (617–652 MHz) & (717–728 MHz) in the other band, and B12, B28, B20 (729–746 MHz & 758–803 MHz & 791–821 MHz) in the third band for later uses. This structure covers the gains from −12 to 18 dB in 6 modes with 6 dB gain steps and with different current consumption. Phase of different gain modes (positive and negative) is usually set inside the modem. However, in this work to achieve ±10${}^{\circ }$ phase discontinuity in CMOS level, instead of a bypass mode for zero and minus gains, we have applied the second path of Common Source (CS) with degeneration. Nevertheless, to keep the area small, the degeneration and load inductors have been shared between these paths.

To achieve the power consumption specifications, the bias voltages of the LNA is controlled. Additionally, to get the Process, Voltage, and Temperature (PVT) independency, a bias voltage compensator together with a Low Drop-Out (LDO) regulator have been applied in the design.

The structure of the paper is as follows: Section 2 discusses the structure of the LNA. In Section 3, the structure of the voltage generator is presented. Section 4 summarizes the experimental results and finally, Section 5 concludes the paper.

## 2. Structure of the Designed LNA

**Figure 2:**
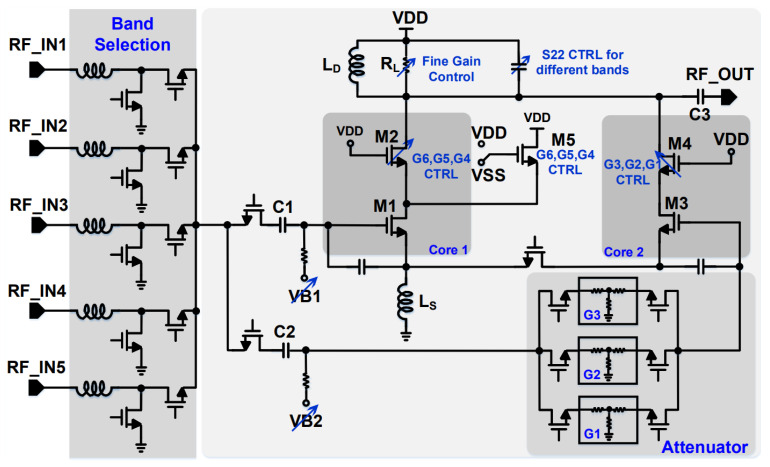
Structure of the designed low band LNA.

Figure 2 shows the structure of the designed low band LNA. To achieve a Noise Figure (NF) below 2 dB at high gains, the core LNA adopts a cascode CS with a source inductive degeneration structure. Ignoring the pad and bonding wire effects, gate-drain capacitance (C_GD_), etc., the NF and gain of the cascode CS LNA at an input resonance frequency can be simplified as below [4]:

(1) $$NF=1+g_{m1}R_{s}\gamma {\left(\omega_{0}/\omega_{T}\right)}^{2}$$

(2) $$V_{out}/V_{in}=R_{L}/\left(2L_{S}\omega_{0}\right)$$

where *g*_*m*1_ is the transconductance of the M1 transistor, *R_s_* is the source impedance, γ is the coefficient where the value is around 2/3 to 1, and *R_L_* and *L_S_* are the load resistance and source inductance values, respectively.

The size of M1 and M2 transistors are 100 μm/60 nm and 50 μm/60 nm, respectively. The G6, G5, and G4 gain modes are being decided in Core 1 on Figure 2, where the gain ranges are 16–19 dB, 10–13 dB, and 4–7 dB, respectively. The supply voltage which is generated by an LDO regulator, is 1 V and the design current specification values for these gain modes are 12 mA, 6 mA, and 3 mA, respectively. Therefore, to control the current in different gain modes, VB1 is being controlled. The VB1 values for G6, G5, and G4 gain modes are 420 mV, 360 mV, and 310 mV, respectively. Reduction of current and V_GS_-V_th_ simultaneously, would reduce the variation of the *g*_*m*_ of the input transistor which is necessary to keep the input matching variation small. In addition, to keep the linearity of the structure in the specification range, it is necessary that the current is not limited by transistor M2, which would cause the V_DS_ of M1 to be reduced and consequently the linearity of the structure to be degraded.

For G6, the gain mode M5 transistor is off and all of the M2 transistors are on. To reduce the gain in G5 and G4 gain modes, M5 turns on and some parts of the transistors in M2, turns off. Then M5 provides some part of the current passing from M1 which helps for the gain reduction. The gain steps are decided according to the ratio of the M5 and M2 transistors [4]. R_L_ also provides fine gain tuning for the designed structure around the gain of the interest. The low gain modes are G3 (−2 to 1 dB), G2 (−8 to −5), and G1 (−14 to −11), which are decided using M3 and M4 transistors where their sizes are 20 μm/60 nm and 20 μm/60 nm, respectively. The VB2 voltage is 420 mV for G3, G2, and G1 gain modes. In order to improve the third-order intermodulation intercept point (IIP3), the attenuation circuit has been applied at the input part of the second path. The design specification defines the current consumption of the G3, G2, and G1 gain modes to be 1.2 mA.

Phase discontinuity is the phase difference when there is a change in the gain modes of the structure, where it should be limited to some specific values. This can be controlled coarsely in Modem. However, for this design, we have implemented the block in such a way that this specification is achievable in analog block level with more accuracy. The reason to apply attenuation modes through active path is to keep the phase discontinuity within ±10${}^{\circ }$. Furthermore, the source degeneration inductance (L_S_) as well as the output load devices have been shared between the first and second paths, and for all of the gain modes in the same band, there will be no major output load changes (for example in the output capacitors). This keeps the phase variation for all gain modes very small.

As mentioned before, this structure is meant to be applied for different frequency bands. To select the different bands, a 5PST switch has been applied at input of the LNA. The transistors of these switches have been stacked to handle the Absolute Maximum Rating (AMR) condition which is 25 dBm power at the RF_INx port of Figure 2. The loss of this switch in the simulation level for all frequencies below 1 GHz remains below 0.2 dB. For different frequency bands, the output capacitors are coarse-tuned to keep the S_22_ in the desired level for the corresponding band. The band selection and tuning are controlled using digital circuitary applied for the whole chip.

## 3. Structure of the Voltage Generator

**Figure 3:**
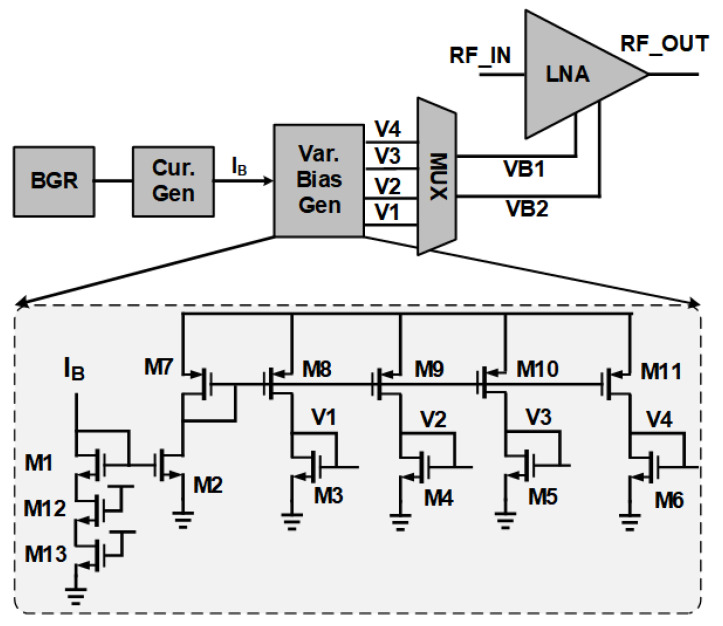
A digitally controllable bias voltage generator.

In order to keep the design independent of PVT variations, the supply voltage is supported by an LDO. The structure of the LDO can be found in [5]. Furthermore, a digitally controllable bias voltage generator has been designed and presented in Figure 3 [6]. This structure provides all of the required bias voltage levels for VB1 and also VB2 for the different gain modes. These voltages can be selected and changed automatically when a change in the gain modes is required. Another intrinsic feature of this block is that for different corner cases, the voltage level changes automatically. With ideal bias voltage, for example for a slow-slow case, the current reduces which consequently degrades other characteristics of the LNA, such as S_11_. However, the designed bias generator automatically increases the bias voltage of the input transistor to compensate for the current reduction and increases the degraded current to its original value and keeps the performance in the required range.

## 4. Experimental Results

**Figure 4:**
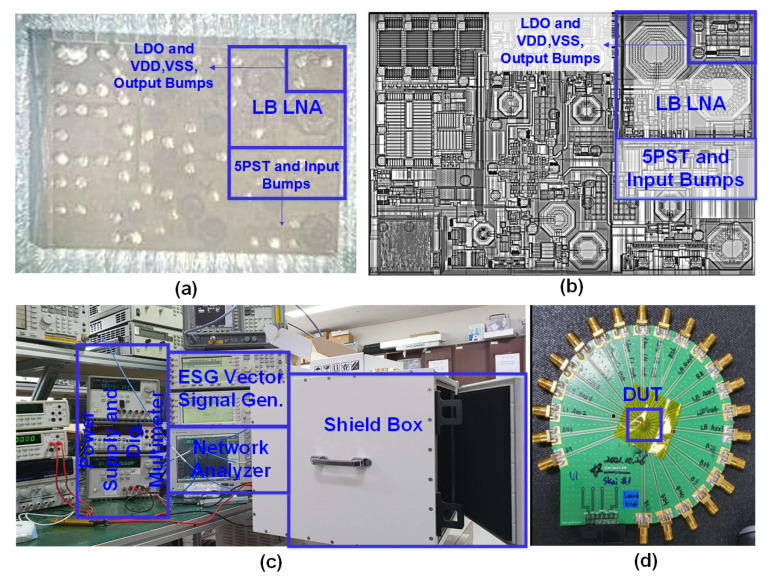
(**a**) Chip micro-photograph, (**b**) layout, (**c**) measurement environment, (**d**) PCB and device under test, of the designed low band LNA as a part of diversity RF front-end for 2G/3G/4G/5G multi-mode cellular handsets.

The presented multi-gain, multi-band LNA has been implemented and measured using a 65-nm RSB process. The chip micro-photograph and layout of the whole system as well as LB LNA have been shown in Figure 4a,b, respectively. Figure 4c presents the measurement environment where vector signal generators, shield box, power supply, digital multi-meter, network analyzer, spectrum analyzer, have been applied for measurements. The Printed Circuit Board (PCB) including device under test has been presented in Figure 4d. The die area of the LB LNA including the core LNA, switches, and bumps is 950 μm × 800 μm. For the measurements, supply voltage is 1 V (LDO output voltage) and the current consumption from G6 to G1 are measured to be 10.8, 6.7, 3.2, 1.3, 1.2, and 1.2 mA, respectively.

**Figure 5:**
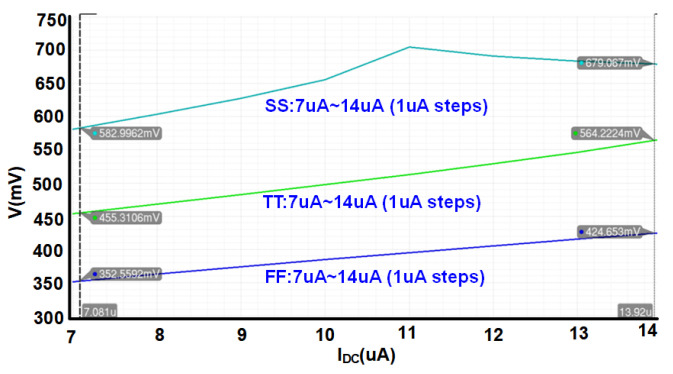
Simulation results of the digitally controllable bias voltage generator for an example voltage, in different corner cases and with different I_B_ reference currents.

Figure 5 presents the simulation results of the digitally controllable bias voltage generator for an example voltage in different corner cases and with different I_B_ reference currents which can be controlled digitally. As we can see, compared to TT corner, for FF, and SS corners, the values of voltage in an specific reference current, are lower and higher respectively. Furthermore, the value can be controlled by sweeping the I_B_ reference current in each corner. As discussed before, this is to control the current of core LNA in different circumstances.

**Figure 6:**
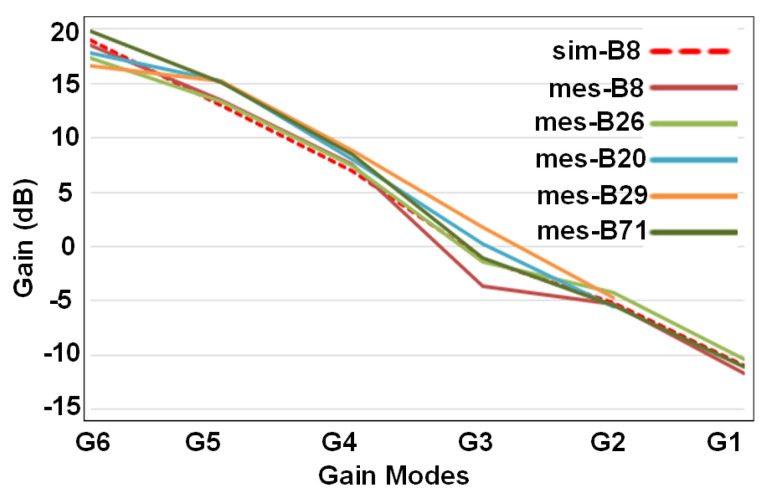
Simulated and measured gain modes for different bands.

**Figure 7:**
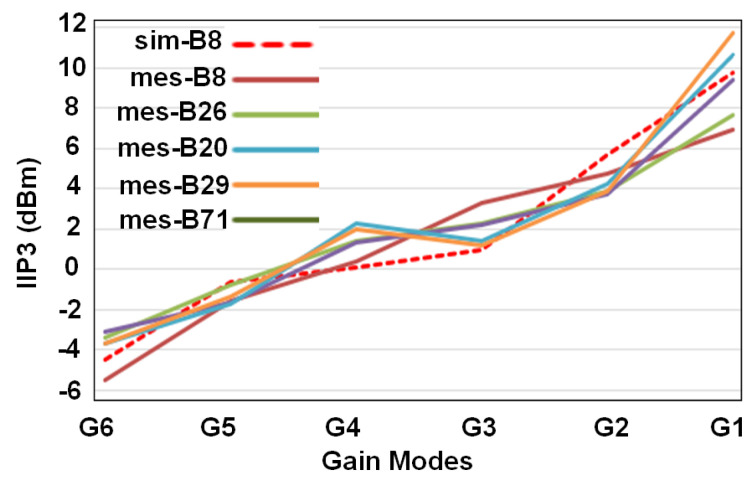
Simulated and measured IIP3 for different bands.

**Figure 8:**
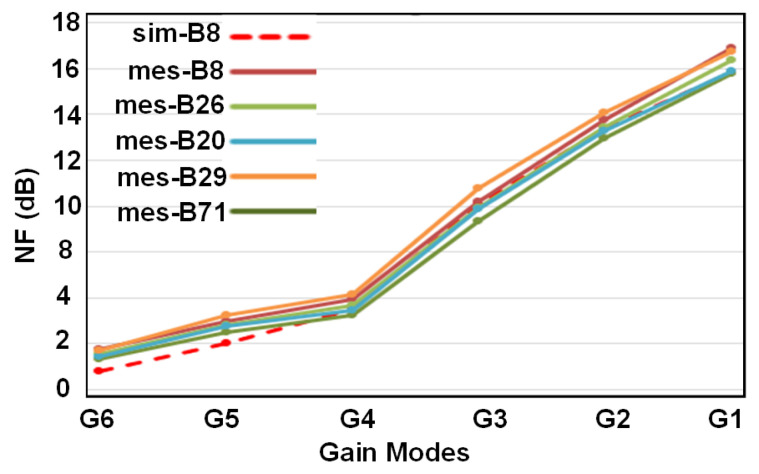
Simulated and measured noise figure for different bands.

Figure 6 shows the simulated G6 to G1 gain-modes for band 8. This figure also incorporates the measured gains for different gain modes in different frequency bands. In the same way, the IIP3 performance of different gain modes and different bands as well as simulated IIP3 for band 8 have been summarized in Figure 7. The best IIP3 has been achieved for G1 mode (the lowest gain) which is almost 12 dBm. Noise figure performance for all gain modes and different frequency bands has been presented in Figure 8. For G6 (the highest gain), the NF remains below 1.9 dB with the best value of 1.2 dB for some cases.

**Figure 9:**
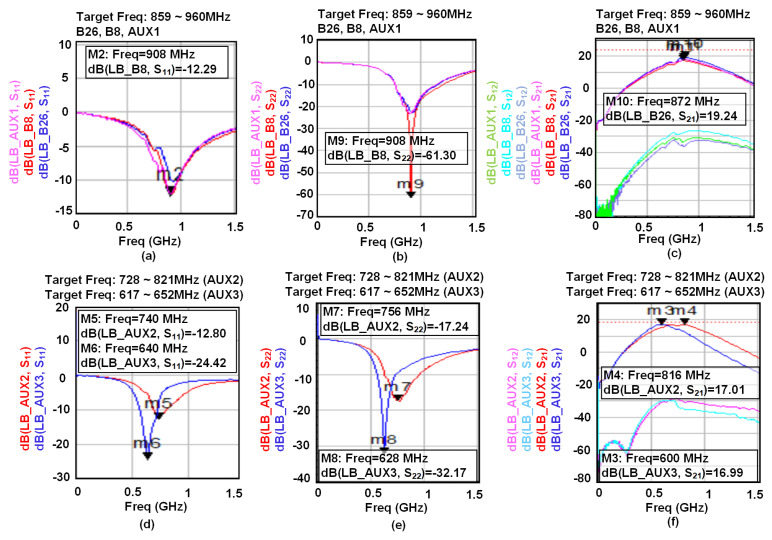
Measured S-parameter results: (**a**) S_11_, (**b**) S_22_, and (**c**) S_12_/S_21_ for 859–960 MHz frequency ranges from B26, B8, and LB_AUX1 ports (Figure 1), (**d**) S_11_, (**e**) S_22_, and (**f**) S_12_/S_21_ for 728–821 MHz and 617–652 MHz frequency ranges from LB_AUX2 and LB_AUX3 ports (Figure 1).

Figure 9 illustrates the S-parameter measurement results for different bands in G6 mode. Figure 9a presents the S_11_ results for the frequency range of 859–960 MHz and through B26, B8, and LB_AUX1 ports shown in Figure 1. In the same way S_22_, S_12_, and S_21_ measurement results have been presented in Figure 9b,c. The S-parameter measurements for frequency ranges of 728–821 MHz and 617–652 MHz have been conducted through LB_AUX2 and LB_AUX3 ports and the results have been summarized in Figure 9d–f. These results (as well as all the presented results) include the Human Body Model (HBM), Charge Device Model (CDM), Electrostatic Discharge (ESD), protections as well as the 5PST switch.

**Figure 10:**
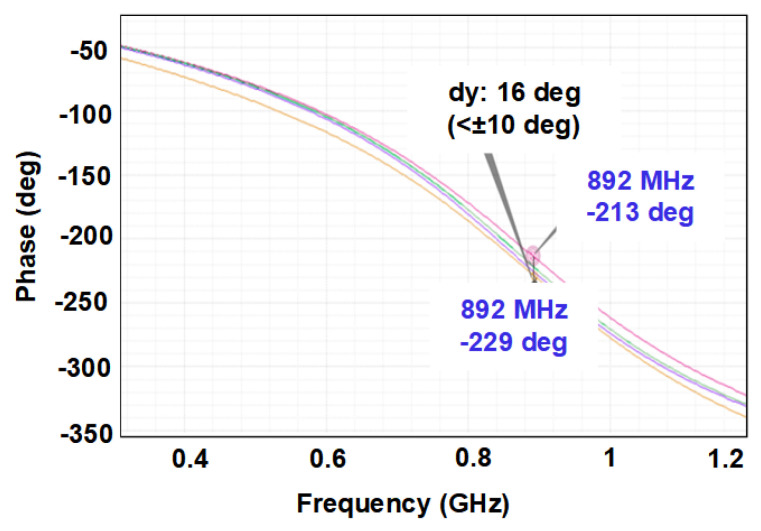
Phase discontinuity in an example frequency of 892 MHz.

The phase discontinuity between the different gain-modes has been also verified which remains below ±10${}^{\circ }$ for the signals in the same frequency band. An example in 892 MHz is shown in Figure 10.

Table 1 summarizes the performance and compares it with other works [7,8,9,10,11,12]. As we can see, our work has a wide gain range (−12∼18 dB) while covering all the frequency range from 617∼960 MHz. Furthermore, both HBM and CDM ESD protections have been applied for this design. This can be considered the reason for the degraded S_11_ performance compared to other presented works. This degradation happens for the G5 and G4 gain modes. Otherwise, for other gain modes this value remains below −10 dB. Finally, this structure shows a better noise figure performance than other presented works except [8]. Nevertheless, we should consider the fact that our structure covers multi-bands with a 5PST switch and the performance of this switch has also been integrated with our proposed LNA.

## 5. Conclusions

This paper presented a low-band low noise amplifier design for a diversity receive module for 2G/3G/4G/5G multi-mode cellular handsets which covered the frequency ranges between 617 MHz to 960 MHz in five different frequency bands. The presented structure covered the gain modes from −12 to 18 dB with 6-dB gain steps, where each gain mode had different current consumption. In order to satisfy the NF specifications in high gain modes, a cascode CS with a source inductive degeneration structure adopted for this design. To achieve the S_11_ parameters and current consumption specifications, the core and cascode transistors for high gain modes (18 dB, 12 dB, and 6 dB) and low gain modes (0 dB, −6 dB, and −12 dB) were separated. Nevertheless, to keep the area low and keep the phase discontinuity within ±10${}^{\circ }$, the degeneration and load inductors were shared between two cores. To compensate the performance for PVT variations, the structure applied an LDO regulator and a corner case voltage compensator. The design was proceeded in a 65 nm RSB PDK and the supply voltage was 1 V. For 18 dB and −12 dB gain modes as two examples, the NF, current consumption, and IIP3 values are 1.2 dB and 16 dB, 10.8 mA and 1.2 mA, and −6 dBm and 8 dBm, respectively.

## Figures and Tables

**Table 1:** Performance summary and comparison.

Parameter	This Work	[7]	[8]	[9]	[10]	[11]	[12]
Freq. (GHz)	0.61–0.96	0.9	0.868/0.9	0.45/0.9	0.05–1	4–11.5	0.3–3.5
NF (dB)	1.2∼1.8	2.2	0.92/0.98	<4.5/3.7	2.3∼3.3	>2.75	>2.9
S_21_ (dB)	−12∼18	11.3	14.2/13.8	>50/30	24∼30	21	14.6
S_11_ (dB)	<−8	−20.6	−18/−16	<−10/−6	<−10	<−10	<−10
S_22_ (dB)	<−15	−23.9	>−15	<−15/−10	NA	NA	NA
IIP3 (dBm)	−6 @ G6	-	−12	−64	−4.1	6.5	1.2
Power (mW)	10.8	12.78	5.23	0.96	19.8	5.15	14.8
ESD	HBM, CDM	NA	HBM	Yes	NA	NA	NA
Multi-Gain	Yes	No	No	Yes	No	No	No
Tech. (nm)	65 (RSB)	180	130	180	65	65	180
